# Clinical and Neuropsychological Correlates of Prefrailty Syndrome

**DOI:** 10.3389/fmed.2020.609359

**Published:** 2020-11-09

**Authors:** Laura Lorenzo-López, Julia Blanco-Fandiño, Nuria Cibeira, Ana Buján, Rocío López-López, Ana Maseda, José Carlos Millán-Calenti

**Affiliations:** Universidade da Coruña, Gerontology and Geriatrics Research Group, Instituto de Investigación Biomédica de A Coruña (INIBIC), Complexo Hospitalario Universitario de A Coruña (CHUAC), Servizo Galego de Saúde (SERGAS), A Coruña, Spain

**Keywords:** aging, prefrailty, neuropsychologial assessment, gait speed, grip strength

## Abstract

Physical frailty is closely associated with cognitive impairment. We aim to investigate the neuropsychological profiles of prefrail and non-frail dementia-free community-dwelling older adults using a comprehensive neuropsychological evaluation, and to examine the association between specific frailty criteria and clinical and neuropsychological scores. Participants completed a comprehensive standardized neuropsychological evaluation (covering cognitive domains such as memory, executive functions, language and attention), and frailty assessment. Frailty was assessed according to biological criteria: unintentional weight loss, exhaustion, low physical activity, slowness, and weakness. The sample comprised 60 dementia-free community-dwelling adults, aged 65 years or older (range 65–89 years; 60.0% women). Forty-two participants were classified as robust (no frailty criteria present), and 18 as prefrail (1 or 2 frailty criteria present). We explored neurocognitive differences between the groups and examined the association between specific criteria of frailty phenotype and clinical and neuropsychological outcomes with bivariate tests and multivariate models. Prefrail participants showed poorer cognitive performance than non-frail participants in both memory and non-memory cognitive domains. However, delayed episodic memory was the only cognitive subdomain that remained significant after controlling for age, gender, and educational level. Gait speed was significantly associated with general cognitive performance, immediate memory, and processing speed, while grip strength was associated with visual episodic memory and visuoconstructive abilities. Both gait speed and grip strength were negatively associated with depressive scores. Our results suggest that prefrailty is associated with cognitive dysfunction. The fact that specific cognitive domains may be susceptible to subclinical states of physical frailty may have important clinical implications. Indeed, early detection of specific cognitive dysfunctions may allow opportunities for reversibility.

## Introduction

Frailty is a common clinical syndrome in older adults due to age-related cumulative decline in multiple physiological systems, associated with negative health outcomes, including deterioration of daily living activities, disability, institutionalization, morbidity, and mortality (1). Cross-sectional and longitudinal research has demonstrated that older adults with physical frailty exhibit poorer cognitive performance and greater risk of cognitive decline and dementia than individuals without frailty (2–15), and that individuals with cognitive decline or dementia show a higher risk of physical frailty (16–18). It has also been demonstrated that the coexistence of physical frailty and cognitive impairment (cognitive frailty), ranging from 1 to 5% in community-dwelling older adults (19), increases the risk of mortality (20–25), functional disability (26), and incident neurocognitive disorders (9, 27) in later life. Importantly, the rates of change of frailty and cognition over time are strongly correlated and associated with the same brain pathologies, such as the presence of macroinfarcts, Alzheimer's disease pathology, and nigral neuronal loss (28). Thus, consideration of both factors is required for the identification of vulnerable older adults at risk of adverse health outcomes (20, 26). With increasing life expectancy, frailty and cognitive disorders, which are increasingly prevalent with advancing age, are being recognized as major healthcare priorities.

Although a growing number of studies are focusing on the relationship between physical frailty and cognitive impairment, most of the researches adopted only a measure of general cognitive functioning to assess cognition or a limited number of cognitive tests (2, 3, 9, 12, 14, 21, 26, 29–35). Moreover, few previous studies have focused on the relationship between prefrailty and cognitive function (35–39), with conflicting results. Some studies reported that prefrailty was not associated with poor cognitive performances (36) and others revealed significant poor performances in several cognitive domains (35, 37–39), but it is unclear whether prefrailty is associated with memory or non-memory cognitive domains, or both. Prefrailty is an intermediate and potentially reversible state between non-frailty and frailty, which has been considered an optimal target for preventive interventions. It has been demonstrated that cognitive performance progressively declines across the continuum from robustness to frailty (40) and that prefrail individuals present more prominent neuroimaging evidence of diffuse cortical or subcortical brain damage than non-frail individuals (41). Because it is unclear whether the associations between frailty and cognitive function are different according to the level of frailty, it is important to identify the cognitive characteristics of prefrailty status. In the present paper, we investigated the neuropsychological profiles of prefrail and non-frail dementia-free community-dwelling older adults using a comprehensive neuropsychological evaluation to identify the cognitive domains most altered by prefrailty. We also examined the association between specific components of frailty phenotype and clinical and neuropsychological scores. Exploring specific cognitive functions and describing neuropsychological correlates of prefrailty may shed light on the understanding of the syndrome, contributing to the investigation of cognitive frailty, and may help to implement intervention strategies for prevention and treatment.

## Materials and Methods

### Participants

This study used a cross-sectional design. Participants were recruited from community centers for older adults located in Galicia (Northwestern Spain), and through distribution of informative leaflets and emails. Talks were given at the centers explaining the purpose, procedures, and assessments to be carried out. Participants were invited to participate in the project and were involved in a voluntary basis. Ninety-one community-dwelling older adults were assessed for eligibility, of which 60 participants without dementia diagnosis (aged 65–89 years, mean age 72.5 ± 6.1 years, 60.0% females) were included in the study and analyzed (18 met the criteria for prefrailty). All participants were 65 years and older, right-handed, free of major physical or psychiatric conditions, and reporting having normal or corrected-to-normal vision and hearing. Participants were excluded from the study if they were taking psychoactive medications based on prescribed medication history, including antipsychotics, antiepileptics, and antidepressants, which could affect cognitive functioning. Institutional review board approval was obtained from the Autonomic Research Ethics of Galicia Committee, Spain (code 2018/049), and research was conducted consistent with the Declaration of Helsinki. Written informed consent was obtained from all participants, who were individually assessed by qualified professionals trained in clinical evaluation.

### Sociodemographic and Clinical Characteristics

The sociodemographic characteristics analyzed were age, gender, and education. Clinical data were collected, including weight and height. Body mass index (BMI) was calculated as weight in kilograms (kg) divided by the square of height in meters (m). Depressive symptoms were determined by the Spanish version of the Geriatric Depression Scale-15 items (GDS-SF) (42, 43).

### Assessment of Frailty

Participants were screened for physical frailty using Fried's criteria (1), including unintentional weight loss ≥ 4.5 kg of body weight in the last year; self-reported exhaustion; low muscle strength (JAMAR hand-grip hydraulic dynamometer), adjusted for gender and BMI; reduced walking speed, assessed by the time (in seconds) needed to walk a distance of 4.57 m, adjusted for gender and height; and low physical activity level, measured by the amount of weekly energy spent (in kilocalories), stratified by gender. Cut-off points of the standardized version of the Fried's criteria were used according to the phenotypic characteristics of the Spanish population (44). Participants who met 1–2 of these criteria were allocated into the prefrail group.

### Neuropsychological Assessment

A neuropsychological battery of tests was administered. The battery involved the assessment of global cognitive function using the Spanish version (45) of the Mini-Mental State Examination (MMSE) (46), and the Montreal Cognitive Assessment test (MoCA) (47). Specific cognitive domains were also explored: episodic memory, attention, executive functioning, naming, and visuospatial function. To assess episodic memory, the immediate memory subscale of the Luria battery for Neuropsychological Diagnosis of Adults was used (Luria-NDA) (48). Visual episodic memory and visuoconstructive abilities were assessed with the Benton Visual Retention Test (BVRT) (49) administration A (immediate recall) and D (short-term retention, with a 15-s interval between the encoding phase and the visual stimulus reproduction), recording the number of correctly reproduced visual stimulus. Visual scanning, psychomotor speed, divided attention, and cognitive shifting were assessed with the Trail Making Test (TMT-A and B) (50). TMT-A is a measure of simple attention and speeded processing and TMT-B is a speeded measure of cognitive flexibility and executive functioning. The time taken to complete each part of the test was recorded in seconds. Visual confrontation naming function was evaluated using the short 15-item version of the Boston Naming Test (BNT) (51), recording the number of correct responses, and excluding perseverations and intrusion errors. Finally, to assess attentional function, the attentional control subscale of the Luria battery for Neuropsychological Diagnosis of Adults (Luria-NDA) (48) was used.

### Statistical Analysis

All variables were checked for normality with the Shapiro-Wilk test. Non-normal continuous dependent variables are reported as median (interquartile range, IQR), normal continuous variables are presented as mean and standard deviations (*SD*), and categorical variables are expressed as count (percentage). A Mann-Whitney *U*-test was conducted to compare differences between groups in the non-normally distributed continuous variables, and an independent *t*-test was conducted for the variables distributed normally. We reported Cohen's r or d effect (52, 53) for the Mann-Whitney and *t*-tests, respectively (0.1 to <0.3 small, 0.3 to <0.5 medium, and 0.5 to 1 large effect sizes). For categorical variables, we used the chi-squared (χ^2^) test. We performed multivariate linear regression models to further explore the relationship between frailty and neuropsychological scores while controlling for demographics (age, gender, and education), as these variables have been previously identified as risk factors for cognitive impairment. Specifically, we estimated separate models for neuropsychological scores that were significant according to univariate analyses, in which the cognitive performance was the dependent variable and a dichotomous variable for frailty (non-frailty and prefrailty) was entered as an independent variable with the covariates. Spearman's Rho partial correlation coefficients adjusted by age were used to assess the relationship between neuropsychological test performance and performance-based frailty criteria; gait speed and hand-grip strength, for the whole sample. These two variables were used as continuous variables to analyze their correlation with cognitive function. Specifically, the time (in seconds) taken to walk 4.57 m at the usual pace was converted to gait speed (meters per second, m/s). Regarding hand-grip strength, three successive readings were taken from the dominant hand in the standardized position, and the highest values (measured in kg) were used in the correlation analysis. Statistical analyses were performed with the statistical software IBM SPSS Statistics, version 25.0. The statistic level of significance was set as *p* < 0.05.

## Results

Of the 60 participants, 18 (30.0%) meet the criteria for prefrailty and 42 (70.0%) did not meet any frailty criteria. Table 1 displays the sociodemographic characteristics and cognitive scores of prefrail and non-frail groups. Participants had a mean age of 72.5 years (*SD* = 6.1), and 34 (60.0%) were females. Prefrail participants were slightly older than robust participants (*p* = 0.049, Cohen's *r* = −0.25). No significant differences in years of formal education (*p* = 0.092) or gender (*p* = 0.206) were observed. Although no significant differences were observed between groups in overall cognitive performance assessed by the MMSE (*U* = 345.5, *z* = −0.542, *p* = 0.588), bivariate tests indicated that prefrail participants showed worse scores in Benton Visual Retention Test, both in immediate (*U* = 216.5, *z* = −2.537, *p* = 0.011, *r* = −0.33) and delayed (*U* = 167.5, *z* = −3.297, *p* = 0.001, *r* = −0.43) applications, poor scores on the Luria-NDA immediate memory subscale (*U* = 226.0, *z* = −2.453, *p* = 0.014, *r* = −0.32), and worse scores in part B of Trail Making Test (*U* = 183.0, *z* = −2.646, *p* = 0.008, *r* = −0.34), compared to non-frail participants. Significant differences were found between the groups in the MoCA scores (*p* = 0.032, *r* = −0.28), with worse scores in the prefrail group. As shown in the Table 1, no significant differences were observed between prefrail and non-frail groups in the naming function (BNT), visual scanning (TMT-A), and attentional control (Luria-NDA- attentional control). Multivariate regression models revealed that delayed episodic memory was the only cognitive subdomain that remained significantly associated with frail status after controlling for age, gender and educational level (see Table 2).

**Table 1:** Clinical characteristics and neuropsychological performance of participants according to their frailty status.

	**Non-frail Group (*n* = 42)**	**Prefrail Group (*n* = 18)**	**Statistics**	***p*-value**
Age (years), median (IQR)	69.00 (66.00–76.25)	73.50 (71.00–79.00)	*z* = −1.969	0.049^*^
Gender, *n* (%)			χ^2^ = 1.601	0.206
Female	19 (45.2%)	5 (27.8%)		
Male	23 (54.8%)	13 (72.2%)		
Education (years), mean (*SD*)	12.67 (5.67)	10.33 (3.48)	*t* = 1.716	0.092
Educational level, *n* (%)			χ^2^ = 2.292	0.318
≤8 years	12 (28.6%)	6 (33.3%)		
9–17 years	21 (50.0%)	11 (61.1%)		
>17 years	9 (21.4%)	1 (5.6%)		
Weight (kg), mean (*SD*)	74.30 (11.88)	71.17 (12.24)	*t* = 0.927	0.358
Height (m), mean (*SD*)	1.62 (0.09)	1.59 (0.08)	*t* = 1.001	0.321
BMI (kg/m^2^), median (IQR)	27.85 (25.52–31.08)	28.03 (26.12–30.73)	*z* = −0.048	0.961
Gait speed (m/s), mean (*SD*)	1.16 (0.23)	1.01 (0.27)	*t* = 2.107	0.039^*^
Hand-grip (kg), median (IQR)	21.50 (15.00–32.00)	12.00 (10.75–22.00)	*z* = −3.393	0.001^*^
GDS score, median (IQR)	1.00 (0.00–2.75)	2.00 (1.00–5.00)	*z* = −1.717	0.086
MMSE score, median (IQR)	29.00 (27.75–29.00)	29.00 (28.00–29.00)	*z* = −0.542	0.588
MoCA score, median (IQR)	25.00 (21.00–27.00)	23.00 (16.00–26.00)	*z* = −2.145	0.032^*^
Luria NDA-AC, median (IQR)	20.87 (19.00–22.00)	20.00 (18.19–21.56)	*z* = −1.300	0.194
Luria NDA-IM, median (IQR)	28.00 (23.50–31.50)	24.25 (16.75–28.50)	*z* = −2.453	0.014^*^
BVRT-A, median (IQR)	5.00 (4.00–7.00)	4.00 (2.75–5.00)	*z* = −2.537	0.011^*^
BVRT-D, median (IQR)	5.00 (4.00–7.00)	4.00 (1.75–4.25)	*z* = −3.297	0.001^*^
BNT, median (IQR)	12.00 (9.00–14.00)	11.00 (8.50–13.00)	*z* = −1.340	0.180
TMT-A (s), median (IQR)	48.03 (38.53–60.99)	56.09 (45.86–76.41)	*z* = −1.629	0.103
TMT-B (s), median (IQR)	113.09 (85.71–146.47)	133.32 (107.83–252.39)	*z* = −2.646	0.008^*^

*Variables distributed non-normally or normally are presented as median (IQR) or mean (SD). BMI, Body Mass Index; BVRT-A, Benton Visual Retention Test, part A; BVRT-D, Benton Visual Retention Test, part D; GDS-SF, Geriatric Depression Scale; MMSE, Mini-Mental State Examination; MoCA, Montreal Cognitive Assessment; NDA-AC, Luria Battery for the Neuropsychological Diagnosis of Adults, Attentional Control; NDA-IM, Luria Battery for the Neuropsychological Diagnosis of Adults, Immediate Memory; TMT-A, Trail Making Test, form A; TMT-B, Trail Making Test, form B*.

* *p < 0.05*.

**Table 2:** Effect of frailty status on neuropsychological scores.

	**MoCA score**		**Luria NDA-IM**		**BVRT-A**		**BVRT-D**		**TMT-B**	
	**β**	***p***	**β**	***p***	**β**	***p***	**β**	***p***	**β**	***p***
Frailty status										
Unadjusted	**−0.278**	**0.031**	**−0.304**	**0.018**	**−0.340**	**0.008**	**−0.475**	**<0.001**	**0.418**	**0.001**
Adjusted^*^	−0.097	0.392	−0.116	0.270	−0.149	0.170	**−0.259**	**0.016**	0.199	0.069

* *Models adjusted for age, gender and education. Statistically significant values are indicated in bold*.

*β, Standardized regression coefficient. BVRT-A, Benton Visual Retention Test, part A; BVRT-D, Benton Visual Retention Test, part D; MoCA, Montreal Cognitive Assessment; NDA-IM, Luria Battery for the Neuropsychological Diagnosis of Adults, Immediate Memory; TMT-B, Trail Making Test, form B*.

**Figure 1:**
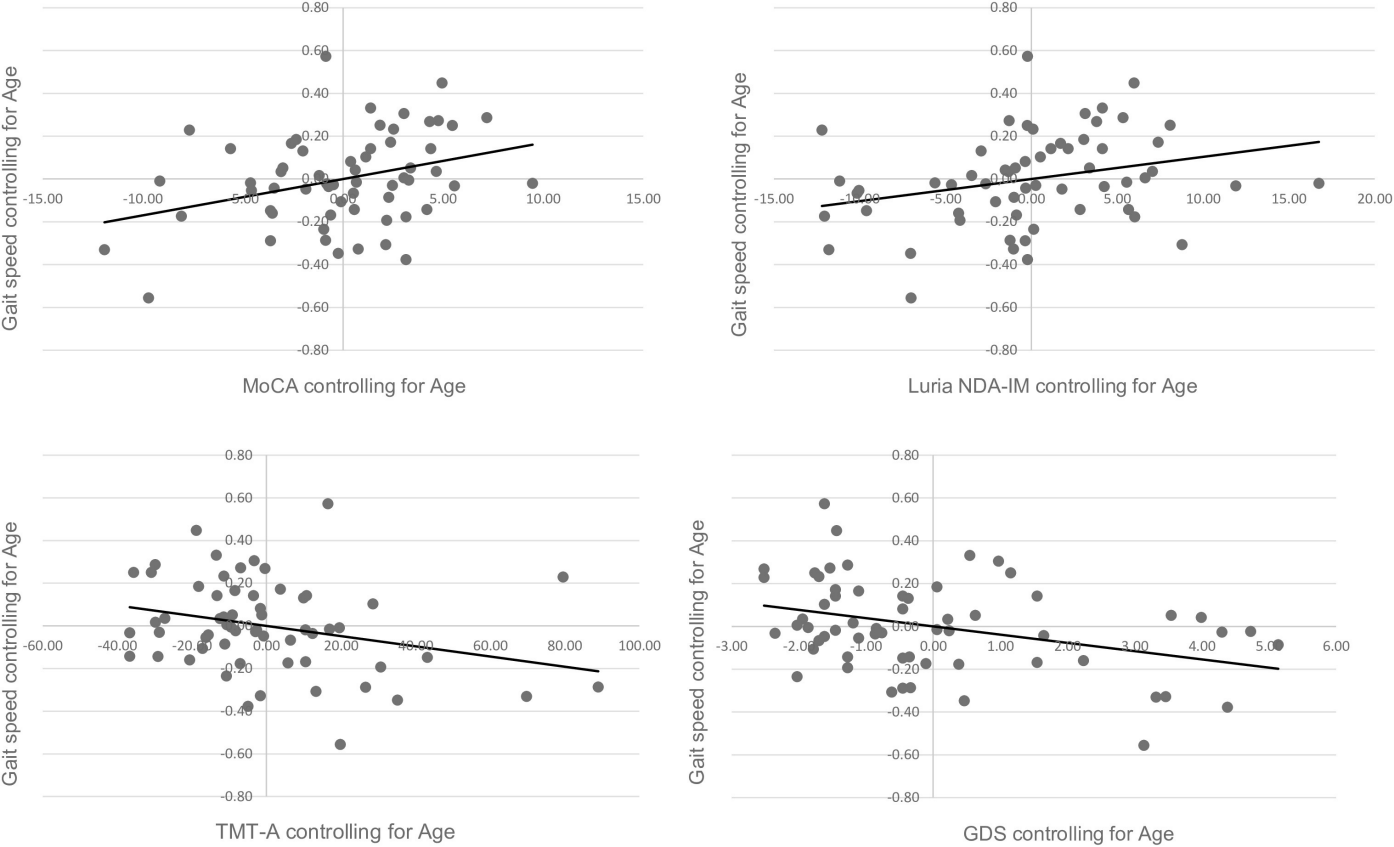
Scatter plots showing the partial correlation between gait speed (m/s) and clinical and cognitive performance, controlled for age. Values on the x-axis represent residuals from regressing MoCA scores, Luria NDA-IM scores, TMT-A scores, and GDS scores on age, respectively. Values on the y-axis represent residuals from regressing gait speed on age.

**Figure 2:**
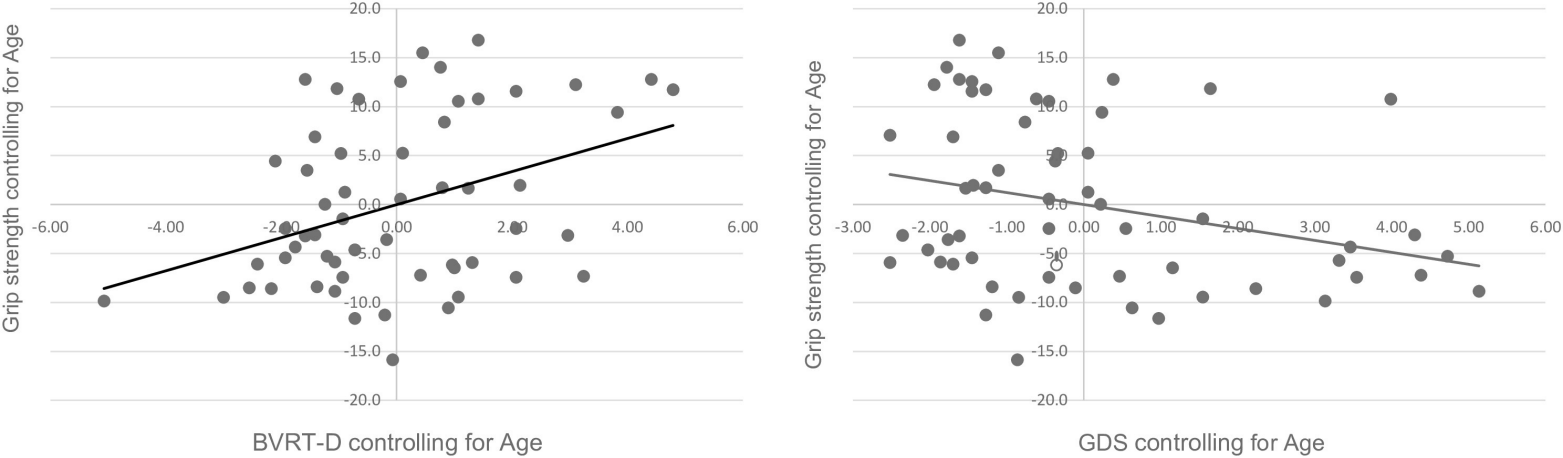
Scatter plots showing the partial correlation between hand-grip strength (kg) and clinical and cognitive performance, controlled for age. Values on the x-axis represent residuals from regressing BVRT-D scores, and GDS scores on age, respectively. Values on the y-axis represent residuals from regressing grip strength on age.

Figures 1, 2 present the scatter plots showing the results of partial correlation coefficients adjusted by age between the gait speed and hand-grip strength, and the clinical and neuropsychological tests, respectively. Rho coefficients (ρ) indexed positive lineal correlations between gait speed (m/s) and cognitive performance in the MoCA test (ρ = 0.258, *p* = 0.048), the Luria-NDA immediate memory subscale (ρ = 0.286, *p* = 0.028), and negative correlations between gait speed and TMT-A (ρ = −0.270, *p* = 0.039) scores. Negative values of the ρ in TMT scores indicate a negative correlation because these scores reflect the time to complete the task and a higher time indicates worse performance. Importantly, gait speed was negatively correlated with GDS-SF scores (ρ = −0.376, *p* = 0.003). Hand-grip strength (kg) was positively correlated with the delayed BVRT (ρ = 0.322, *p* = 0.015) and negatively correlated with GDS-SF scores (ρ = −0.343, *p* = 0.008).

## Discussion

The primary aim of the present study was to examine neuropsychological correlates of prefrailty in dementia-free community-dwelling older adults and to examine the association between specific components of the frailty phenotype and clinical and neuropsychological performance.

### Neuropsychological Correlates of Prefrailty

Our findings associate prefrailty with the presence of specific neuropsychological impairments. Indeed, in the simple comparisons prefrail participants demonstrated significantly poorer cognitive functions, including executive function, verbal and visual immediate memory, and visuospatial function than non-frail participants with the same level of education. It is important to note, however, that only visual episodic memory domain remained significantly associated with frailty status when controlling for demographic characteristics. In recent research, poor results on delayed memory and processing speed were observed in prefrail older adults with cognitive complaints (38). It has been previously shown that prefrail older adults with no diagnosis of cognitive impairment present poor global cognitive function than non-frail older adults (30, 37). In a study exploring the neuropsychological profiles of cognitive frailty, it was observed that cognitively frail individuals had significant impairments in memory and visuospatial domains than those with cognitive impairment with no physical frailty (54). Comorbid prefrailty and cognitive impairment have been previously associated with future frailty and mortality (21, 35), and with an increased risk for dementia (9).

The poor performance on the BVRT, which assess episodic visual memory, reveals potential different patterns of visual scanning and fixation in the prefrail participants. Poorer performances (longer times) in the TMT-B may suggest problems in divided attention and cognitive shifting in prefrail participants. However, this difference was not significant after controlling for demographics. This is a speeded measure of cognitive flexibility involving mental tracking and switching between letter-number sets, evaluating the executive function. A significant correlation between frailty and TMT-B was previously observed (55). Previous studies have also reported a significant impairment of sustained attention (56) and executive function in prefrail (39) and frail (55, 57–60) older adults.

No significant differences were observed between prefrail and non-frail groups in the naming function, assessed by the BNT, in the present work, in contrast to previous studies (39, 61). Because prefrailty is an intermediate state of frailty syndrome, some authors (61) hypothesized that language impairment could contribute to a more rapid progression of the syndrome. Because no relationship was found between frailty state and performance on language tests in our study (40, 60), our results do not support this hypothesis (61).

No significant differences were found between the groups in attentional control assessed by the Luria-NDA subscale. This subscale includes five items assessing selective and sustained attention function. This finding suggests that the mechanisms of attentional control seem preserved in prefrail older adults.

As stated in the introduction, in the literature it is unclear whether prefrailty status is associated with memory or non-memory cognitive domains, or both. Some previous cross-sectional studies focusing on prefrailty, revealed differences in both memory and non-memory cognitive domains (37, 38), and other study found differences only in non-memory domains (39). In general, our findings provide evidence of the susceptibility of the memory cognitive domain to prefrailty status in relatively healthy participants with the same level of education and free of dementia or cognitive complaints. Although we also observed poor performance in other non-memory cognitive domains, adjustments diminished the statistical significance other than delayed visual memory. Thus, our findings contradict studies showing that non-memory domains seem to be influenced first in the prefrail status (39). Importantly, it has been pointed out that not all cognitive subdomains may become impaired simultaneously but may be impaired depending on the specific frailty criteria present and age (37), and this may partially explain the differences among studies. To our knowledge, only one longitudinal study investigated the effects of combinations of cognitive impairment and prefrailty on cognitive outcomes (35), revealing that prefrail participants with cognitive impairments (cognitive prefrailty) had poorer delayed recall at 4-years follow-up. However, only a measure of general cognitive status was used in this study (35). Given the inconsistencies in the results, future longitudinal studies are needed to further explore the association between prefrailty and cognition including neuroimaging findings. Identifying cognitive differences between non-frail and prefrail older adults will be useful for future intervention studies, assisting in the establishment of optimal multimodal strategies.

It is important to note that no significant differences in global cognition evaluated by the MMSE were observed between prefrail and non-frail groups in the present study. However, cognitive differences between the groups were sensitively captured by using the MoCA. This finding suggests that there may be cognitive performance problems related to frailty that are not detectable by the global measurement of the MMSE alone. In this sense, efforts to detect and further understand frailty should include a consistent measurement of specific cognitive domains employing comprehensive neuropsychological testing.

### Association Between Specific Frailty Criteria and Neuropsychological Performance

Individual criteria of physical frailty have been previously associated with cognition. In the present paper, we explored the association between objectively measured physical capacity criteria (low grip strength and slow gait speed) and cognitive performance, because they are more prevalent than physiological markers (self-reported exhaustion, unintentional weight loss, and low physical activity) in the prefrail Galician population (62), and they have shown a better ability to predict future disability (63). Moreover, the components most strongly associated with cognitive function among older adults are grip strength and gait speed (6, 17, 34, 37, 39, 64, 65), and weakness and slowness have been shown the first emerging components of physical frailty (66). The rates of change over time of both gait speed and grip strength is strongly correlated with the rate of change in cognition (28). Slow gait speed is a predictor of transitions between mild and severe cognitive decline and mortality (67). Both motor skills (gait and grip strength) contributing to physical frailty and cognition depends on the integrity of the central nervous system (68). A recent neuroimaging study revealed a significant association between gait speed and brain amyloid-β accumulation in the temporal cortex, parietal cortex, precuneus/posterior cingulate cortex, and basal ganglia, and a significant association between weakness and a general brain amyloid-β accumulation (69).

Self-reported fatigue or exhaustion has been also significantly associated with poor global cognition (30, 37) and a higher risk of incident mild cognitive impairment (6) and dementia (33).

The present study demonstrated an association between gait speed and general cognitive performance assessed by the MoCA, immediate memory, and processing speed. Slower gait speed has been previously associated with worse scores in attention, executive function (37), memory tests (70), slow processing speed (37, 38, 71, 72), and verbal fluency (34, 72). Importantly, the combination of slow gait speed and cognitive impairment has been associated with a high risk for progression to dementia (32). However, a more regular and predictable gait pattern, but not gait speed, was previously correlated with cognitive decline in other studies (73). Performance on the TMT-B has been previously associated with performance on usual gait speed tests in older adults with cognitive impairment (74). In our study, gait speed was negatively associated with the performance on the TMT-A reflecting processing speed, but not in the TMT-B associated with executive functions.

Hand-grip strength was positively correlated with visual episodic memory and visuoconstructive abilities in the present study. Low hand-grip strength has been previously associated with reduced cognitive performance over time (68), and with a higher risk of developing mild cognitive impairment (6). Grip-strength has been associated with performance on the MMSE (34, 75), and executive function (37). In a longitudinal study, grip strength performance was associated with a change in verbal ability, spatial ability, processing speed and memory after age 65 years (76). Weakness was the most common initial manifestation in prefrail women (66). Because gender-related differences may exist in both grip-strength and cognitive function, future studies focusing in these differences are needed to get a better understanding of their association.

Our findings may have specific clinical implications since hand-grip is a simple and modifiable factor that can be useful for the monitoring of the progression of cognitive impairment (68).

Finally, it is important to note that in our study, both gait speed and grip strength negatively correlated with depressive symptomatology (GDS-SF score). The relationship between physical frailty and depression has been previously reported (33, 77, 78). It has been shown that frail depressed older adults show worse performance than non-frail depressed in speed-dependent executive functions and verbal fluency (79), and that the severity of physical frailty was associated with poor verbal memory, slower processing speed and decreased working memory (71). These findings suggest that depression seems to be an important condition to take into account when disentangling the association between prefrailty and cognitive impairment. Thus, future research is needed to further explore the mechanisms underlying associations among physical frailty, cognitive dysfunction, and depressive symptoms. Future work should also explore the longitudinal relationships between hand-grip strength and gait speed and cognitive performance.

The main strengths of this preliminary study were the comprehensive neuropsychological evaluation and the focus on the prefrailty status. The main limitation, however, was the small sample size, which may increase the variability of the data and reduce the statistical power. The cross-sectional design and the slight age difference between the groups limited our ability to interpret the cause-effect relationship of the association between physical prefrailty and cognitive performance, since cognitive changes occur over the life span. Future longitudinal studies including comprehensive neuropsychological testing and neuroimaging information are needed to further explore the dynamic nature of both frailty/prefrailty and cognition and the influence of genetic and environmental factors in their relationship.

Our findings confirm that memory cognitive domain may be susceptible to a subclinical state of physical frailty or prefrailty. Knowledge about specific cognitive deficits associated with prefrailty is important as such markers may help early identification of persons at risk of frailty and dementia. We conclude that the comprehensive assessment for cognitive impairment may be effective for identifying prefrail older adults at higher risk of frailty, dementia, and mortality. This may have important clinical implications since prefrail older adults with cognitive impairments are targets for preventive interventions. Both physical and cognitive therapy should be recommended for the prevention and treatment of frailty.

## Data Availability Statement

The raw data supporting the conclusions of this article will be made available by the authors, without undue reservation.

## Ethics Statement

The studies involving human participants were reviewed and approved by Autonomic Research Ethics of Galicia Committee, Spain (code 2018/049). The patients/participants provided their written informed consent to participate in this study.

## Author Contributions

LL-L and JM-C: study concept and design. AB, RL-L, NC, and JB-F: acquisition of data. LL-L, AB, and JB-F: analysis and interpretation of data. LL-L and JB-F: drafting of the manuscript. LL-L, AB, AM, JB-F, RL-L, NC, and JM-C: critical revision of the manuscript for important intellectual content. All authors: contributed to the article and approved the submitted version.

## Conflict of Interest

The authors declare that the research was conducted in the absence of any commercial or financial relationships that could be construed as a potential conflict of interest.
